# Imaging of inferior vena cava normal variants, anomalies and pathologies, Part 1: Congenital

**DOI:** 10.4102/sajr.v27i1.2687

**Published:** 2023-11-24

**Authors:** Ranjit K. Chaudhary, Pankaj Nepal, Shruti Kumar, Elina Gupta, Nikita Sangroula, Arpit Nagar, Vijayanadh Ojili

**Affiliations:** 1 Department of Radiology, St. Vincent’s Medical Center, Bridgeport, United States of America; 2 Department of Radiology, Massachusetts General Hospital, Boston, United States of America; 3 Department of Radiology, University of Arkansas, Little Rock, United States of America; 4 Department of Radiology, Ohio State University Wexner Medical Center, Ohio, United States of America; 5 Department of Radiology, University of Texas Health, San Antonio, United States of America

**Keywords:** IVC, vena cava, IVC anomalies, Azygos continuation, Abernathy malformation, IVC web, IVC embryology

## Abstract

The embryology of the inferior vena cava (IVC) is complex, involving the sequential appearance and regression of multiple segments that ultimately form the IVC. Any alteration in this process during embryogenesis can result in congenital anomalies of the IVC. This study aimed to recognise common as well as rare anomalies of the IVC and associated veins, and their clinical implications. The anomalies tend to have diverse appearances based on the timing and segments involved. The development of the IVC is intertwined with the development of other veins like the renal vein, azygos vein and portal vein, and these veins may also be anomalous. Additionally, IVC anomalies are associated with various other congenital anomalies including cardiac anomalies, the recognition of which may be important for patient care. The IVC tends to have multiple normal variants and anomalies because of a complex process involving multiple segments contributing to the adult IVC. Knowledge of these variants is crucial for preoperative planning of procedures.

**Contribution:**

This study would help in understanding the embryogenesis of the IVC and correlation with the imaging appearances and the clinical implications of each of these common as well as rare types of congenital anomalies.

## Introduction

Congenital anomalies of the inferior vena cava (IVC) are often recognised as incidental findings at abdominal imaging. Most congenital variations are asymptomatic, but knowledge of these abnormalities can be crucial for preoperative planning of surgeries and endovascular procedures, as well as to avoid misdiagnosis.^1^ The embryonic development of the IVC is complex involving numerous sequences in formation, regression, and persistence of the embryonic veins, which eventually contribute to different segments. Any abnormality of this process can lead to an abnormal IVC and anomalous veins. Occasionally, congenital anomalies of the IVC can be associated with congenital cardiac abnormalities, the recognition of which may be crucial for patient management.^2^ This article emphasises the understanding of congenital IVC variants and their clinical significance.

## Anatomy and embryology

The IVC is the main route of venous return from the lower extremities and abdomen, formed by the confluence of the two common iliac veins at L5. It has a retroperitoneal course in the abdomen, located to the right of aorta. It courses close to the liver where it receives the hepatic veins and then passes through the diaphragmatic hiatus at the T8 level to become intrathoracic, terminating in the right atrium.^3^

The four segments of the IVC are the hepatic, suprarenal, renal, and infrarenal segments (Table 1).^4^ The development of the different segments of the IVC is complex and involves the sequential appearance, anastomoses and regression of three pairs of embryonic veins, namely the posterior cardinal, subcardinal, and supracardinal veins.

**TABLE 1:** Embryonic vein and their corresponding adult derivatives.

Embryonic vein	Derivatives
Right vitelline vein	Suprahepatic and hepatic IVC segments
Right subcardinal vein	Suprarenal IVC segment
Right supra-subcardinal and post-subcardinal anastomosis	Renal segment
Right supracardinal vein	Infrarenal segment
Posterior cardinal vein	Iliac veins
Supracardinal veins in thoracic region	Azygos and hemiazygos veins

IVC, inferior vena cava.

During the fourth week of foetal life, the posterior cardinal veins develop and become dominant by the sixth week, responsible for the return of all the blood from the body wall caudal to the heart.^5^ The vitelline vein, which drains the embryonal yolk sac, is responsible for blood return from the viscera and eventually forms the hepatic segment of the IVC. The subcardinal veins become the dominant venous system by the seventh week of foetal life. The posterior cardinal veins are dorsolateral and the aorta is ventromedial to the subcardinal veins. The suprarenal segment of the IVC develops from the right subcardinal vein. By 8 weeks, the supracardinal veins predominate, positioned dorsomedial to the posterior cardinal veins and dorsolateral to the aorta. The intrathoracic course of the supracardinal veins form the azygous and hemiazygos veins. The left supracardinal vein disappears, while the right supracardinal vein forms the infrarenal segment of the IVC. The renal segment of the IVC is formed by the anastomoses between the subcardinal and supracardinal veins.^1,5,6^ The iliac veins derived from the persistent posterior cardinal veins form an anastomosis with the infrarenal IVC derived from the right supracardinal vein.^7^

Although the posterior cardinal veins do not contribute to the adult IVC, abnormality in development of the posterior cardinal veins can lead to IVC anomalies.^5^ Anomalies in this complex process can lead to various congenital anomalies of the IVC, which has been reported in nearly 4% of the general population (Table 2).^2^ Associated congenital abnormalities can occur particularly in cases with azygos continuation of the IVC, which are related to heterotaxy syndrome with left isomerism.^2,8^

**TABLE 2:** Classification of inferior vena cava anomalies.

Anomalous segment	Congenital anomalies
Supracardinal veins	Left-sided IVC Double IVC
Postcardinal vein	Retrocaval ureter
Renal segment	Circumaortic and retroaortic renal vein
Subcardinal-hepatic anastomosis	Azygos continuation of the IVC Double IVC retroaortic right renal vein and hemiazygos continuation of IVC Double IVC retroaortic left renal vein and azygos continuation of IVC
Variable segment	Absence of IVC

IVC, inferior vena cava.

The ureter, during development, takes it course posterior to the posterior cardinal veins. The supracardinal vein is posteromedial to the embryonic ureter. The inter-supracardinal anastomosis posteriorly, inter-subcardinal anastomosis and post-subcardinal anastomoses anteriorly, and the supra-subcardinal anastomosis laterally together forms the renal collar. The paired ventral and dorsal limbs drain the kidneys during early development. Both dorsal limbs usually regress, while the ventral limb on right side is incorporated into the lateral wall of the renal segment of the IVC. The ventral limb on left side and the anterior limb of the renal collar form the normal adult left renal vein.^1^

## Imaging

Ultrasonography (US) is a readily available, cheaper and safer imaging modality. Although often used for the initial evaluation of the IVC, particularly in paediatric patients, it has limitations of operator dependence and difficult visualisation because of obscuration by bowel gas.^2,7,9^

Contrast enhanced CT abdomen is the preferred and most commonly performed imaging modality for evaluation of the IVC. The IVC is best evaluated in the venous phase with a delay time of 70 s – 90 s after intravenous contrast administration to allow for uniform enhancement of the infrarenal IVC. However, imaging is commonly performed in the portal venous phase during routine abdominal CT at 60 s – 70 s. Multiplanar reformation can be obtained with multidetector CT, which is often useful in delineating the course of the IVC and anastomosis.^2,7,9,10^

Although MRI has the advantage of not utilising ionising radiation, it is an uncommonly used modality. Its limited role is because of high cost, need for anaesthesia in the paediatric population, and limited availability. Evaluation of the IVC can be performed using post-contrast three-dimensional breath-hold T1-weighted MRI. Balanced steady-state free precession is another useful MRI sequence for imaging of the IVC.^7,9^ Magnetic resonance venography (MRV) can be performed using the time of flight (TOF) or phase contrast technique, which does not require administration of gadolinium contrast.^11,12^

## Congenital anomalies

Most congenital anomalies are asymptomatic, but their identification is necessary to avoid mistaking them for pathology and for planning of vascular procedures (Table 3).^13^ Most of the congenital IVC abnormalities, particularly those that result in an abnormal vein or abnormal dilation of normal veins, can mimic lymphadenopathy.^1^

**TABLE 3:** Congenital variants and abnormalities of inferior vena cava.

Developmental variations	Prevalence	Vein regresses	Vein persists	Course	Clinical significance
Normal IVC	-	L supracardinal V	R supracardinal V	Normal	Normal
L sided IVC	0.2% – 0.5%	R supracardinal V	L supracardinal V	L IVC ends at L renal V and crosses ventral to the aorta to form a normal suprarenal IVC.	Difficult transjugular IVC filter placement. Spontaneous rupture of AAA into L IVC.
Double IVC	1% – 3%	-	Both supracardinal V	L IVC crosses anterior to the aorta to join the R IVC.	Recurrent PE despite IVC filter placement.
Circumaortic L renal V	2.4% – 8.7%	-	Both inter-supracardinal and inter-subcardinal V	One L renal V crosses anterior and another posterior to the aorta.	Preoperative planning for nephrectomy. Challenging renal venous sampling.
Retroaortic L renal V	1.7% – 3.4%	Ventral arch of inter-subcardinal anastomosis	Dorsal arch of the renal collar	L renal V passes posterior to the aorta.	Preoperative planning.
Circumcaval or retrocaval ureter	-	R supracardinal V	R posterior cardinal V	Proximal ureter courses posterior to the IVC.	Increased risk of ureteral obstruction or recurrent UTI.
Azygos continuation of the IVC	0.6%	R subcardinal-hepatic anastomosis and R subcardinal V	-	Suprarenal IVC continues cranially as azygos V.	Preoperative planning of cardiopulmonary bypass. Associated anomalies.
Double IVC with Retroaortic R Renal V and Hemiazygos continuation of IVC	Rare	R subcardinal-hepatic anastomosis ventral arch of R inter-subcardinal anastomosis	L lumbar and thoracic supracardinal V and L supra-subcardinal anastomosis R dorsal limb of renal collar	R renal V and R IVC join and cross aorta posteriorly to join L IVC, continues cephalad as hemiazygos V to join rudimentary azygos V but mostly drains into collaterals.	Aberrant vessels may simulate L mediastinal mass or aortic dissection.
Double IVC with retroaortic L renal V & azygos continuation of IVC	Rare	R subcardinal-hepatic anastomosis ventral arch of L inter-subcardinal anastomosis	L supracardinal V and dorsal limb of renal collar	R renal A abnormally crosses anterior to the IVC. Double IVC draining into prominent azygos.	Prominent vein can mimic mass. Challenges in IVC filter placement.
Absence of IVC	Extremely rare	Varies depending on segment of IVC absent	-	-	Prominent collaterals may mimic paraspinal mass.
Abernathy malformation	Extremely rare	Excessive involution or failure of vitelline V to establish anastomosis with hepatic sinusoids or hepatic V	-	Shunting of portal blood into IVC. Absent portal vein in type I.	Associated with FNH, HCC hepatic encephalopathy and bleeding varices. Type I associated with congenital cardiac, GI and GU anomalies.

IVC, inferior vena cava; R, right; L, left; V, vein; A, artery; UTI, urinary tract infection; FNH, focal nodular hyperplasia; HCC, hepatocellular carcinoma; GI; gastrointestinal; GU, genitourinary; AAA, abdominal aortic aneurysm; PE, pulmonary embolism.

### Left-sided inferior vena cava

**FIGURE 1:**
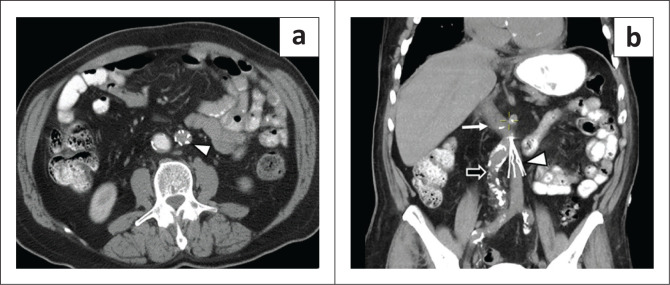
A 53-year-old male with left lower quadrant pain was found to have acute diverticulitis with abscess formation. (a) Contrast enhanced axial CT abdomen image showed a left-sided inferior vena cava (IVC) with a filter (arrowhead). Knowledge of the anatomy was mandatory prior to IVC filter placement in this case. (b) Coronal CT abdomen of same patient shows the IVC to the left of the atherosclerotic aorta (open arrow). The left IVC then turns towards right at the level of left renal vein (not shown) to continue as the normal right suprarenal IVC (white thin arrow).

A left-sided IVC is formed as a result of disappearance of the right supracardinal vein and persistence of the left supracardinal vein. The left-sided IVC typically terminates at the left renal vein. It then courses ventral to the aorta to the right side to form a normal right-sided suprarenal IVC (Figure 1). The reported incidence is 0.2% – 0.5%.^1,14,15^ It is a mirror image variant without an increased risk of any abnormality. However, it can lead to confusion between venous and arterial access. Also, transjugular IVC filter placement and pulmonary thrombolysis is more challenging with a left-sided IVC. In addition, it can mimic left paraaortic adenopathy.^1,9^ Rare spontaneous rupture of an abdominal aortic aneurysm into a left IVC has also been reported.^16,17^

### Double inferior vena cava

**FIGURE 2:**
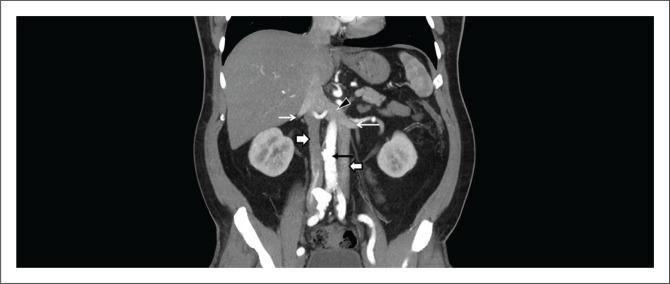
A 31-year-old female with acute right lower quadrant pain related to a haemorrhagic cyst. Contrast enhanced coronal CT image demonstrated duplication of the inferior vena cava (IVC) (white thick arrows) with the aorta (black thin arrow) in between. The renal veins (white thin arrows) are draining into the ipsilateral IVC. The left IVC after joining the left renal vein courses to the right to communicate (black arrowhead) with the right IVC.

A double IVC has a left-sided IVC in addition to the normal right IVC. It results from persistence of both supracardinal veins. The left IVC crosses the midline anterior to the aorta to join the right IVC (Figure 2). There may be variations in calibre of the left and right IVC.^1,9^ The incidence of double IVC has been reported between 1% and 3%.^15^ Recurrent pulmonary embolism despite IVC filter placement should raise suspicion of a double IVC. Review of prior cross-sectional images before IVC filter placement is useful to diagnose congenital IVC abnormalities such as double IVC. If no prior cross-sectional images are available for review, cavography through the left iliac vein should be performed prior to filter placement. A filter can be placed into each cava if a double IVC is present. The aberrant vessel may also mimic a lymph node.^1,7,9^

### Circumaortic left renal vein

**FIGURE 3:**
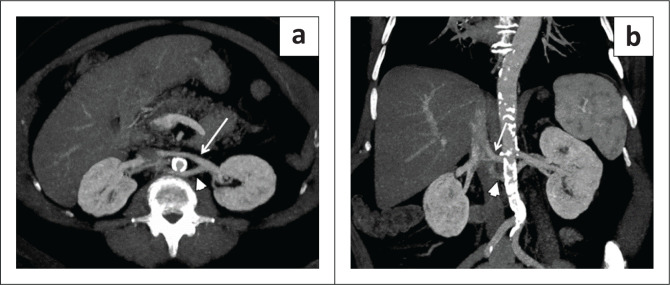
A 61-year-old man who underwent preoperative imaging of the chest, abdomen and pelvis with contrast enhanced CT for aortic valve replacement. (a) Axial CT image demonstrated two left renal veins, one crossing anterior to the aorta (arrow) and the second (arrowhead) coursing posterior to the aorta. (b) Coronal CT multiplanar reconstruction image demonstrated two left renal veins with an inferior location of the retroaortic renal vein (arrowhead).

Two left renal veins are present on the left side with one coursing anterior to the aorta and other posterior to the aorta. It results from persistence of the posteriorly located inter-supracardinal vein anastomoses as well as the anteriorly located inter-subcardinal vein anastomoses that form a venous ring around the aorta (Figure 3). The reported prevalence ranges from 2.4% to 8.7%.^1^ The posterior renal vein is 1 cm – 2 cm inferior to the normal anterior vein. The left adrenal vein drains into the superior renal vein. The left gonadal vein drains into the inferior renal vein. Knowledge of this variation is significant in preoperative planning prior to nephrectomy and catheterisation for renal venous sampling. It can also mimic retroperitoneal adenopathy.^7,9^ It is also important to recognise this variation prior to positioning of the IVC filter.^18^ These patients may uncommonly present with hypertension, haematuria and varicoceles.^19^

### Retroaortic left renal vein

A single left renal vein with a retroaortic course as the dorsal arch of the renal collar persists, while the ventral arch (inter-subcardinal anastomosis) regresses. Reported prevalence is 1.7% – 3.4%.^1,7^ Although mostly asymptomatic, occasionally patients may present with symptoms of haematuria, flank pain and varicocele.^19,20^

### Retrocaval or circumcaval ureter

**FIGURE 4:**
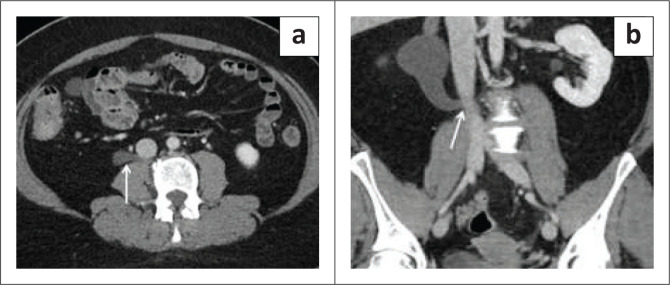
A 47-year-old female with urinary tract infection and haematuria demonstrated right-sided hydronephrosis on ultrasound. Axial post-contrast CT (a) and coronal (b) images demonstrate a retrocaval course of the ureter (arrow).

The genitourinary system develops separately from the IVC. However, embryogenesis of the IVC determines the spatial relationship between the ureter and the IVC. In this anatomical variant, the infrarenal segment develops from the right posterior cardinal vein, which is anterolateral to the ureter, instead of the right supracardinal vein, which is posteromedial to the ureter. The anomaly almost always occurs on the right side with a handful of cases reported on left.^9,21^ The reported prevalence of a retrocaval ureter is 0.13%.^22^ In intravenous urography, the proximal part of retrocaval ureter makes a characteristic course. It projects over or medial to the lumbar pedicles. This gives the characteristic fish hook or reverse J appearance (Figure 4).^7,9,19^ Although most cases are asymptomatic, right flank pain is the most common symptom.^21^ Compression of the ureter between the IVC and vertebra can result in hydronephrosis. Ureteral obstruction or recurrent urinary tract infection (UTI) may necessitate surgical relocation of the ureter anterior to the cava.^9,19,21^ Coexistence of other congenital genitourinary, cardiovascular and spine abnormalities have been reported.^21^

### Interruption of the inferior vena cava with azygos or hemiazygos continuation

**FIGURE 5:**
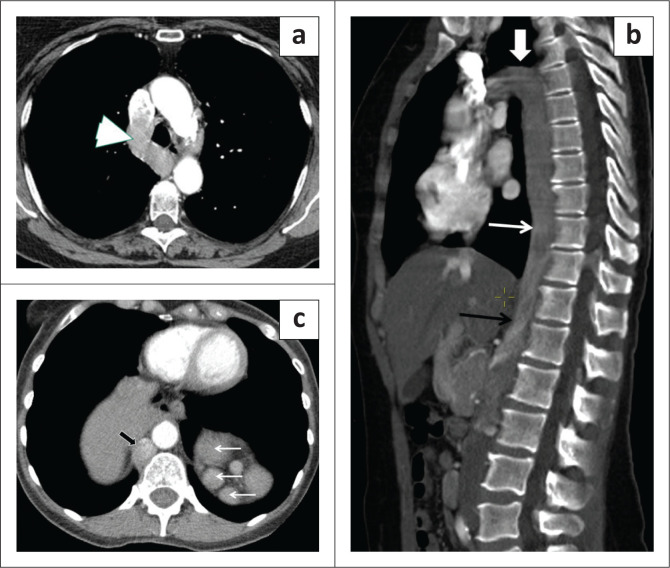
A 61-year-old female with a history of a colorectal mass on colonoscopy underwent CT chest, abdomen and pelvis for staging. (a) Post-contrast axial CT image shows a dilated azygos vein. (b) Sagittal oblique CT image shows the inferior vena cava (IVC) continuing into the thorax as the dilated azygos vein (white thin arrow) and arching (white thick arrow) in the superior mediastinum to drain into the superior vena cava. The black arrow represents the site of mixing of denser contrast from the renal vein. (c) Axial CT image at the level of aortic hiatus demonstrates azygos continuation of the IVC (black arrow) with polysplenia (white arrows) and mesocardia. The patient also had a midline liver and intestinal malrotation (not shown) suggestive of a left isomerism type of heterotaxy syndrome.

Absence of the hepatic segment of the IVC is thought to be because of failure of the right subcardinal-hepatic anastomosis with resultant right subcardinal vein atrophy and shunting of blood from the supra-subcardinal anastomosis to the azygos vein. The azygos vein joins the superior vena cava at the right paratracheal space in the expected normal location. The hepatic segment is not entirely absent. It drains directly into the right atrium. The right gonadal vein drains to the ipsilateral renal vein as the post-subcardinal anastomosis does not contribute to the formation of the IVC. It has a reported prevalence of 0.6%.^1,7,9^ Azygos continuation is more common than hemiazygos continuation. Azygos continuation of the IVC can be seen in asymptomatic patients or in association with other congenital abnormalities such as severe congenital heart disease and asplenia or polysplenia syndromes (Figure 5).^1,8,23^ An enlarged azygos vein can mimic a right paratracheal or retrocrural lymph node. Knowledge of this variation is useful for preoperative planning for cardiopulmonary bypass and vascular procedures.^1,9^

### Double inferior vena cava with retroaortic right renal vein and hemiazygos continuation of the inferior vena cava

This rare anomaly results from anomalies in multiple steps during the development of the IVC and renal vein. Similar to azygos continuation of the IVC, absence of the right subcardinal-hepatic anastomosis results in failure of development of the hepatic segment. Left lumbar and thoracic supracardinal veins persist giving rise to a left IVC and blood is shunted into the hemiazygos vein via the left supra-subcardinal anastomosis. Persistence of the dorsal limb and regression of the ventral limb of the renal collar leads to a right renal vein that meets the right IVC and courses posterior to the aorta to join the left IVC. The azygos vein is rudimentary. The hemiazygos vein drains via alternate collateral pathways. The hemiazygos vein can drain into the azygos vein, the coronary vein of the heart in patients with a persistent left superior vena cava or via the accessory hemiazygos vein into the left brachiocephalic vein. These aberrant vessels may simulate a left mediastinal mass or aortic dissection. Like azygos continuation of the IVC, the hepatic segment of the IVC drains into the right atrium independently.^1,24^

### Double inferior vena cava with a retroaortic left renal vein and azygos continuation of the inferior vena cava

**FIGURE 6:**
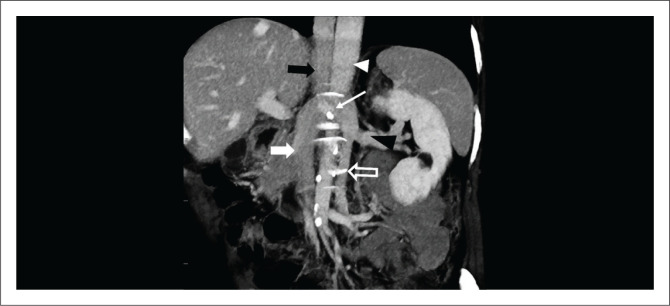
Coronal oblique CT reconstruction of a contrast enhanced abdomen shows a double inferior vena cava (IVC) with left and right IVCs indicated by an open white arrow and a solid white arrow, respectively. The IVC then continues as the azygos vein (black thick arrow) to the right of the aorta (white arrowhead). The left renal vein (black arrowhead) courses posterior to the aorta to the right side to drain into the right IVC as shown by thin white arrow.

This congenital variation occurs when the supracardinal vein and the dorsal limb of the renal collar persist while the ventral limb regresses and the subcardinal-hepatic anastomosis does not form.^1,25^ These are extremely rare and detected incidentally. Very few cases of right-sided varicocele have been reported in patients with a double IVC and azygos or hemiazygos continuation.^25,26^ The imaging findings are similar to azygos continuation of the IVC except there is a double IVC instead of the normal right-sided IVC and the left renal vein has a retroaortic course^25^ (Figure 6). Clinical implications of this variation are to avoid misdiagnosis as lymphadenopathy and preoperative surgical planning.^25^

### The absence of the inferior vena cava

**FIGURE 7:**
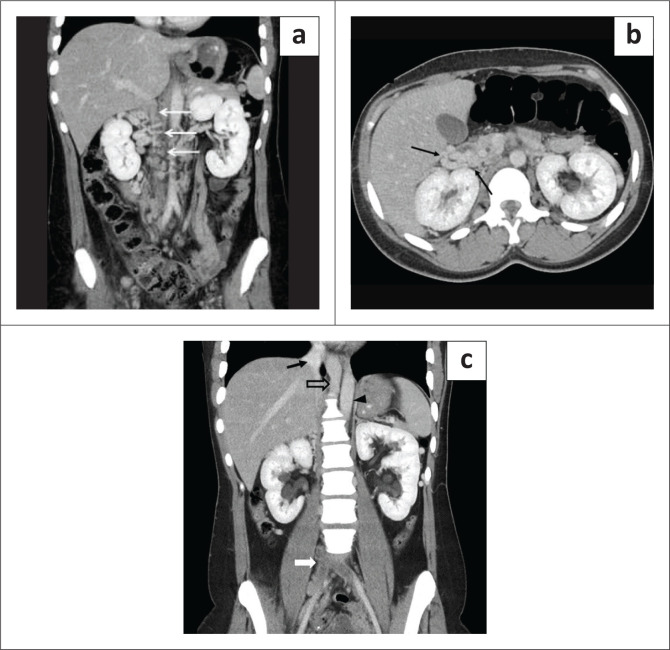
A 54-year-old female with non-specific abdominal pain. (a) Coronal contrast enhanced CT image demonstrated an absent infrarenal inferior vena cava (IVC) with multiple collaterals (thin white arrows). (b) Axial contrast enhanced CT image of the same patient shows non-visualisation of the IVC with multiple collaterals (thin black arrows). (c) Bilateral common iliac veins fuse (thick white arrow) but do not continue as the IVC, draining via multiple collaterals into the azygos vein (open black arrow) seen to the right of aorta (black arrowhead). The suprahepatic IVC (black solid arrow) is present, draining into the right atrium.

The entire IVC or only the infrarenal portion of the IVC can be absent. Absence of the entire posthepatic IVC results from failure of development of all three paired venous systems. These are extremely rare. The absence of the infrarenal IVC implies failure of development of the posterior cardinal and supracardinal veins.^9^ As a single embryonic event cannot explain this abnormality, it is thought that it is not a true embryonic anomaly but the result of perinatal thrombosis and atrophy.^1,7,9^ Imaging shows the absence of the IVC or a portion of the IVC with prominent venous channels providing collateral flow (Figure 7). These veins are prone to idiopathic deep vein thrombosis and lower extremity venous insufficiency. Prominent lumbar collateral vessels may develop, which can mimic paraspinal masses.^9^

The common iliac veins can be absent as well. The lower extremity blood drains to the azygos and hemiazygos veins via anterior paravertebral collateral veins. Anterior paravertebral collateral veins receive blood via enlarged ascending lumbar veins, which in turn are formed by the external and internal iliac veins.^1^

### Extrahepatic portocaval shunt or Abernethy malformation

The portal vein is formed cranially from a segment of the prehepatic right vitelline vein, the intervitelline anastomosis, and caudally from the left vitelline vein around the fifth week of life.^19^ The right vitelline vein thus serves as a common conduit during the development of both the IVC and the portal vein. The process of formation of hepatic tissue results in disruption of intervitelline vein anastomosis disconnecting the cranial and caudal segments of the portal vein. Eventually, the cranial segment of the portal vein disappears. Hence, there is no direct communication between the portal vein and the IVC.^19^ These extrahepatic portocaval shunts may result either from excessive involution of the vitelline vein or failure of the vitelline vein to establish an anastomosis with the hepatic sinusoids or hepatic veins.^9,27^

**FIGURE 8:**
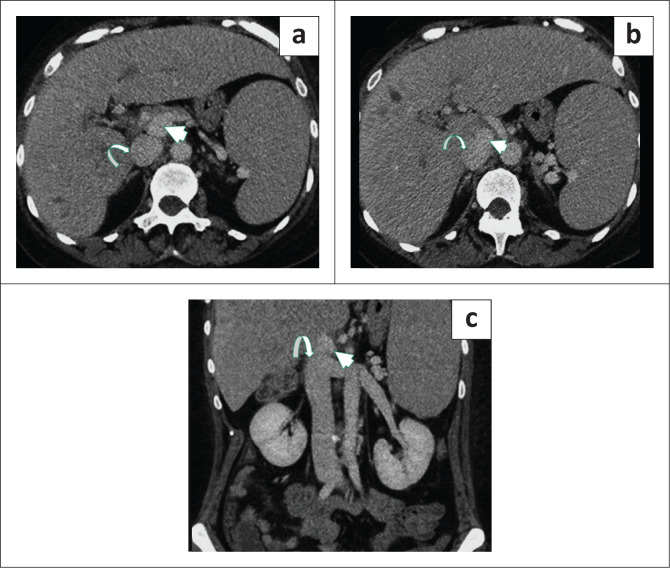
A 48-year-old male patient was imaged for abdominal trauma. Axial post-contrast CT (a, b) and coronal (c) images demonstrated the main portal vein (arrowhead) draining directly into the inferior vena cava (curved arrow). Incidental splenomegaly and mildly dilated intrahepatic bile ducts are also seen.

Abernethy malformations are of two types. Type 1 is more common in females and is characterised by complete shunting of portal blood into the IVC and an absent portal vein (Figure 8). It is associated with congenital cardiac, gastrointestinal (e.g., biliary atresia) and genitourinary anomalies.^9,19^ Type 2 is more often seen in males as an isolated abnormality with partial end-to side anastomosis between an intact portal vein and the IVC resulting in shunting of blood. The presence or absence of the portal vein is an important imaging finding because it helps to distinguish between the two types. The Abernethy malformation is associated with focal nodular hyperplasia and hepatocellular carcinoma (HCC).^7,9^ Type I is managed with a liver transplant. The Type II shunt may require occlusion of the shunt if the patient develops hepatic encephalopathy or bleeding varices. It can be managed surgically or percutaneously using balloons or coils.^19^

### Inferior vena cava membranes and webs

**FIGURE 9:**
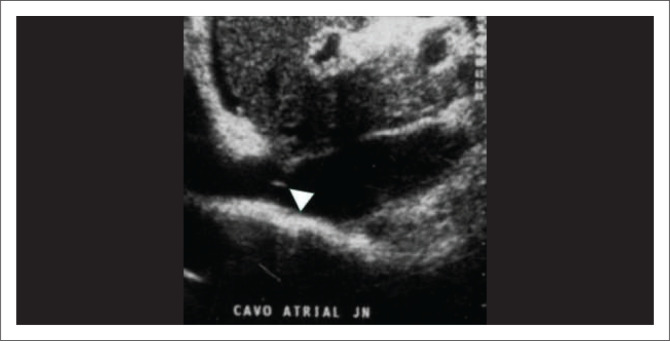
A 34-year-old male who was evaluated for Budd-Chiari syndrome was found to have a web (arrowhead) in the inferior vena cava at the level of cavoatrial junction on ultrasonography.

These are uncommon IVC findings attributed to congenital vascular anomalies or the sequela of thrombus formation.^9^ North American and northern European populations are rarely affected. There is usually an underlying hypercoagulable state and they present acutely. Outcomes are usually fatal. Asian and South African populations are more commonly affected in whom it eventually leads to congestive cirrhosis.^28^ The onset is insidious in these patients.^7,9^ A complete or fenestrated membrane or a segment of fibrotic occlusion in the intrahepatic IVC with prominent intrahepatic and extrahepatic collaterals is seen on imaging (Figure 9). Inferior venacavography can be helpful in confirming the diagnosis. Endovascular treatment to relieve portal pressure can be performed depending on the severity of the associated liver disease.^7,9,29^ The association between membranous obstruction of the intrahepatic IVC, Budd-Chiari syndrome and HCC is well established.^7^

## Conclusion

Congenital anomalies of the IVC are often asymptomatic but may have clinical implications. Knowledge of these variations is crucial in preprocedural planning of IVC filter placement or preoperative planning for cardiovascular surgeries as well as nephrectomy. These congenital anomalies may predispose to certain conditions such as urinary obstruction and UTI. Moreover, some of these anomalies have been associated with other congenital anomalies. Thus, it seems prudent to be cognizant of these IVC variations and ensuring that treating physicians are aware of these findings so that they can plan their treatment appropriately.

## References

[1] Bass JE, Redwine MD, Kramer LA, Huynh PT, Harris Jr JH. Spectrum of congenital anomalies of the inferior vena cava: Cross-sectional imaging findings 1: (CME available in print version and on RSNA Link). Radiographics. 2000;20(3):639–652. 10.1148/radiographics.20.3.g00ma09639 10835118

[2] Sheth S, Fishman EK. Imaging of the inferior vena cava with MDCT. Am J Roentgenol. 2007;189(5):1243–1251. 10.2214/AJR.07.2133 17954667

[3] Agur AM, Dalley AF. Moore’s essential clinical anatomy. Philadelphia, US: Lippincott Williams & Wilkins; 2018.

[4] Li SJ, Lee J, Hall J, Sutherland TR. The inferior vena cava: Anatomical variants and acquired pathologies. Insights Imaging. 2021;12:1–22. 10.1186/s13244-021-01066-7 34460015 PMC8405820

[5] Malaki M, Willis AA, Jones R. Congenital anomalies of the inferior vena cava. Clin Radiol. 2012;67(2):165–171. 10.1016/j.crad.2011.08.006 22070941

[6] Chuang VP, Mena CE, Hoskins PA. Congenital anomalies of the inferior vena cava. Review of embryogenesis and presentation of a simplified classification. Br J Radiol. 1974;47(556):206–213. 10.1259/0007-1285-47-556-206 4824552

[7] Kandpal H, Sharma R, Gamangatti S, Srivastava DN, Vashisht S. Imaging the inferior vena cava: A road less traveled. Radiographics. 2008;28(3):669–689. 10.1148/rg.283075101 18480478

[8] Applegate KE, Goske MJ, Pierce G, Murphy D. Situs revisited: Imaging of the heterotaxy syndrome. Radiographics. 1999;19(4):837–852. 10.1148/radiographics.19.4.g99jl31837 10464794

[9] Smillie RP, Shetty M, Boyer AC, Madrazo B, Jafri SZ. Imaging evaluation of the inferior vena cava. Radiographics. 2015;35(2):578–592. 10.1148/rg.352140136 25763740

[10] Petik B. Inferior vena cava anomalies and variations: Imaging and rare clinical findings. Insights Imaging. 2015;6(6):631–639. 10.1007/s13244-015-0431-z 26373648 PMC4656244

[11] Butty S, Hagspiel KD, Leung DA, Angle JF, Spinosa DJ, Matsumoto AH. Body MR venography. Radiol Clin North Am. 2002;40(4):899–919. 10.1016/S0033-8389(02)00028-3 12171191

[12] Glockner JF, Lee CU. Magnetic resonance venography. Appl Radiol. 2010;39(6):36. 10.37549/AR1758

[13] Eldefrawy A, Arianayagam M, Kanagarajah P, Acosta K, Manoharan M. Anomalies of the inferior vena cava and renal veins and implications for renal surgery. Cent European J Urol. 2011;64(1):4–8. 10.5173/ceju.2011.01.art1 PMC3921701 24578852

[14] Shin DS, Sandstrom CK, Ingraham CR, Monroe EJ, Johnson GE. The inferior vena cava: A pictorial review of embryology, anatomy, pathology, and interventions. Abdom Radiol. 2019;44(7):2511–2527. 10.1007/s00261-019-01988-3 30937506

[15] Phillips E. Embryology, normal anatomy, and anomalies. Venography of the inferior vena cava and its branches Baltimore, MD: Williams & Wilkins, 1969; p. 1–32.

[16] Davachi AA, Thomas J, Dale WA, Perry FA, Michael OB. Acute spontaneous rupture of an arteriosclerotic aneurysm into an isolated left-sided inferior vena cava. Am J Cardiol. 1965;15(3):416–418. 10.1016/0002-9149(65)90340-1 14263043

[17] Niino T, Unosawa S, Shimura K. Ruptured abdominal aortic aneurysm with left-sided inferior vena cava. Ann Vasc Surg. 2012;26(7):1012.e9–1012.e11. 10.1016/j.avsg.2012.02.017 22944577

[18] Hislop S, Fanciullo D, Doyle A, Ellis J, Chandra A, Gillespie DL. Correlation of intravascular ultrasound and computed tomography scan measurements for placement of intravascular ultrasound-guided inferior vena cava filters. J Vasc Surg. 2014;59(4):1066–1072. 10.1016/j.jvs.2013.10.071 24388045

[19] Ghandour A, Partovi S, Karuppasamy K, Rajiah P. Congenital anomalies of the IVC – Embryological perspective and clinical relevance. Cardiovasc Diagn Ther. 2016;6(6):482. 10.21037/cdt.2016.11.18 28123970 PMC5220208

[20] Nam JK, Park SW, Lee SD, Chung MK. The clinical significance of a retroaortic left renal vein. Kor J Urol. 2010;51(4):276–280. 10.4111/kju.2010.51.4.276 PMC2858856 20428432

[21] Perimenis P, Gyftopoulos K, Athanasopoulos A, Pastromas V, Barbalias G. Retrocaval ureter and associated abnormalities. Int Urol Nephrol. 2002;33(1):19–22. 10.1023/A:1014436432109 12090330

[22] Hostiuc S, Rusu MC, Negoi I, Grigoriu M, Hostiuc M. Retrocaval ureter: A meta-analysis of prevalence. Surg Radiol Anat. 2019;41(11):1377–1382. 10.1007/s00276-019-02269-w 31201483

[23] Gayer G, Apter S, Jonas T, et al. Polysplenia syndrome detected in adulthood: Report of eight cases and review of the literature. Abdom Imaging. 1999;24(2):178–184. 10.1007/s002619900471 10024407

[24] Sahin H, Pekcevik Y, Aslaner R. Double inferior vena cava (IVC) with intrahepatic interruption, hemiazygos vein continuation, and intrahepatic venous shunt: A rare variant of IVC anomalies. Vasc Endovascular Surg. 2017;51(1):38–42. 10.1177/1538574416687734 28100158

[25] Ahmetoglu A, Cansu A. Duplication of the inferior vena cava with azygos continuation, retroaortic left renal vein and iliac vein variations. Bratisl Lek Listy. 2012;113(7):448–450. 10.4149/BLL_2012_100 22794522

[26] Madani AH, Mokhtari G, Jandaghi AB, Teimoori M. Right varicocele secondary to leftsided inferior vena cava with a retro-aortic left renal vein and azygos continuation. Turk J Urol. 2019;45(1):73. 10.5152/tud.2018.98853 30668310 PMC6342572

[27] Howard ER, Davenport M. Congenital extrahepatic portocaval shunts – The Abernethy malformation. J Pediatr Surg. 1997;32(3):494–497. 10.1016/S0022-3468(97)90614-X 9094026

[28] Kew M, Hodkinson H. Membranous obstruction of the inferior vena cava and its causal relation to hepatocellular carcinoma. Liver Int. 2006;26(1):1–7. 10.1111/j.1478-3231.2005.01194.x 16420504

[29] Murphy EH, Johns B, Varney E, Raju S. Endovascular management of chronic total occlusions of the inferior vena cava. J Vasc Surg. 2016;63(1):291. 10.1016/j.jvs.2015.10.041 27987609

